# Motional consensus of self-propelled particles

**DOI:** 10.1038/s41598-023-35238-w

**Published:** 2023-05-20

**Authors:** Jia-xin Qian, Jun Wang, Yan-qing Lu

**Affiliations:** 1 National Laboratory of Solid State Microstructures, Collaborative Innovation Center of Advanced Microstructures, Nanjing University, Nanjing, 210093 China; 2 College of Engineering and Applied Sciences, Nanjing University, Nanjing, 210093 China; 3 School of Physics, Nanjing University, Nanjing, 210093 China

**Keywords:** Computational biophysics, Statistical physics

## Abstract

The motional consensus of self-propelled particles is studied in both noise-free cases and cases with noise by the standard Vicsek model. In the absence of noise, we propose a simple method, using grid-based technique and defining the normalized variance of the ratio of the number of particles locally to globally, to quantitatively study the movement pattern of the system by the spatial distribution of the particles and the degree of aggregation of particles. It is found that the weaker correlation of velocity leads to larger degree of aggregation of the particles. In the cases with noise, we quantify the competition between velocity alignment and noise by considering the difference of the variety of order parameter result from the velocity alignment and noise. The variation of the effect of noise on motional consensus is non-monotonic for the change of the probability distribution of noise from uniform to non-uniform. Our results may be useful and encourage further efforts in exploring the basic principles of collective motion.

## Introduction

Collective behavior extensively exists in both the macroscopic systems such as the crowds of human^1^, the herds of mammal^2^, the flocks of bird^3–6^, the schools of fish^7^ and the swarms of insect^8^ and the microscopic systems including bacterial colonies, cells etc^9–12^. Studying the motional consensus of collective behavior is of great importance in discovering the basic principle of collective motion, which enables us to better understand the mechanism of escaping from predators, foraging etc^13,14^. In addition, the study of motional consensus inspires the control of multibody systems such as a number of robotic machines^15^, which is beneficial to exploring efficient motion strategies for escaping from fire etc^16^.

The Vicsek model^17^, proposed in 1995, is one of the classical models for studying collective motion of active systems. It considers the velocity alignment and noise for updating the velocity of all of the particles. Flocking phenomena is observed when the noise is small, which means the motion of all the agents almost reach global alignment and the degree of motional consensus is high. Spontaneously local^18^ and global velocity alignment^19^ also be explored. Because the Vicsek model is simple but catches the main factors of collective motion, many researchers have paid attention to it in theory as well as simulation^20–24^. Series of interesting phenomena are observed including traveling bands^25^, moving crystals^26^, Swirlonic state^27^, phase transition^28–30^ and circular pattern^31,32^. Various factors, including hybrid noise^33^, aggregation interaction^34^, low density and low speed^35^, auditory sensing^36^, view angle^37,38^, chirality^39^ and complex noise environment^40^, are considered for exploring the diverse phenomenon of collective behavior as well. Some model, modifying based on Vicsek model, is proposed to improve the speed of motional consensus by adjusting the rules of velocity alignment such as remote neighbors strategy^41^, updating the direction with exponential weights depending on the neighbor numbers^42,43^ or according to the direction of the neighbors^42,44^.

Besides the progress that has been made in exploring collective behavior, the quantitative description of the movement pattern of the particles and the mechanism for the formation of different movement patterns of standard Vicsek model remain unclear. And how the competition between velocity alignment and noise affects motional consensus is not clear enough. Studying them is important to improving our understanding of the mechanism of collective motion. Therefore, in noise-free cases, we study the movement pattern of motional consensus by the spatial distribution of the particles and the degree of aggregation of the particles. And we proposed a method to quantitatively describe the spatial distribution of the particles. The effect of different parameters on the movement pattern of motional consensus is analyzed and the reason of different movement patterns is explored. As for the effect of velocity alignment and noise on motional consensus, we quantify the competition between them and find the non-monotonic variation of the effect of noise on motional consensus.

## Model

Here, we consider the standard Vicsek model^17^ including *N* self-propelled particles. All of the particles are regarded as points and continuously move in a two-dimensional square cell. The linear size of the square cell is *L* and the cell is considered with periodic boundary conditions. The interaction radius among particles is *r* which means the field of vision for each particle is ${\pi r^2}$. Particles in the field of vision of particle *i*, including particle *i* itself, are regraded as the neighbor of it. The motion of each particle obey the ordinary differential equations (ODEs) as follows

1 $$\begin{aligned} \left\{ \begin{array}{l} \frac{d \mathbf {x_{i}}}{dt} = \mathbf {v_{i}} = ve^{i\theta _{i}t} \quad \\ \frac{d \mathbf {v_{i}}}{dt} = ve^{i\left( \frac{1}{\mathcal {N}_{i}(t)} \sum _{j \in \mathcal {N}_{i}(t)} (Arg \ \mathbf {v_{j}} - Arg \ \mathbf {v_{i}}) + \xi _{\theta _{i}} \right) } \end{array} \right. \end{aligned}$$

where $\mathcal {N}_{i}(t) = \{j : |\mathbf {x_{j}} - \mathbf {x_{i}}| \leqslant r \}$ denotes the number of the neighbor of particle *i* at time *t* and ${\xi _{\theta _{i}}}$ is random noise. And the ODEs of the model in 2D cartesian coordinates are as follows

2 $$\begin{aligned} \left\{ \begin{array}{l} \frac{dx_{i,1}}{dt} = v_{i,1} = v\cos {\theta _{i}} \quad \\ \frac{dx_{i,2}}{dt} = v_{i,2} = v\sin {\theta _{i}} \quad \\ \frac{dv_{i,1}}{dt} = v\cos \left( \frac{1}{\mathcal {N}_{i}(t)} \sum _{j \in \mathcal {N}_{i}(t)} (\theta _{j} - \theta _{i}) + \xi _{\theta _{i},1} \right) \quad \\ \frac{dv_{i,2}}{dt} = v\sin \left( \frac{1}{\mathcal {N}_{i}(t)} \sum _{j \in \mathcal {N}_{i}(t)} (\theta _{j} - \theta _{i}) + \xi _{\theta _{i},2} \right) \end{array} \right. \end{aligned}$$

In the simulations, the time step updating the velocity and position of all of the particles is ${\Delta t = 1}$. The initial positions of all of the particles are randomly distributed in the cell. Using the Euler method to discrete the Eq.(1), simultaneous update of the position of all of the particles at each time step according to

3 $$\begin{aligned} \mathbf {x_{i}}(t+1) = \mathbf {x_{i}}(t) + \mathbf {v_{i}}(t) \Delta t \end{aligned}$$

and *i* denoting different particle among all of the N particles, takes one to *N*. All of the particles have the same absolute value of velocity *v*. The initial direction of velocity of all of the particles are randomly and uniformly distributed in ${[-\pi ,\pi )}$. The variation of the velocity is shown in the change of the direction of the velocity. According to Eq.(1), the rule for updating the direction of velocity of the particles is

4 $$\begin{aligned} \theta _{i}(t+1) = \langle \theta _{i}(t) \rangle _{r} + \Delta \theta \end{aligned}$$

where ${\langle \theta _{i}(t) \rangle _{r}}$ is the average direction of all of the neighbors of the particle *i*, which is given by

5 $$\begin{aligned} { \langle \theta _{i}(t) \rangle _{r} = \arctan \left[ \frac{\langle \sin (\theta _{i}(t)) \rangle _{r}}{\langle \cos (\theta _{i}(t)) \rangle _{r}} \right] } \end{aligned}$$

and ${\Delta \theta }$ represents noise which is a random number following uniform distribution in ${[-\eta /2,\eta /2]}$. ${\eta }$ is the parameter to control the amount of noise.

In order to measure the degree of the motional consensus of the system, the normalized average velocity of all the *N* particles is considered as the order parameter of the system, which is as follows

6 $$\begin{aligned} { \phi = \frac{1}{Nv} \left| \sum _{i = 1}^{N} \mathbf {v_{i}} \right| } \end{aligned}$$

When the direction of the velocity of the particles achieves global alignment (flocking state), normalized average velocity ${\phi }$ reaches 1 and zero in the randomly disordered states^17^.

## Result and discussion

### The noise-free cases

In noise-free cases, ${\eta = 0}$. When the number of simulated time steps is large enough, the order parameter of the system can reach 1. This means that the system arrives in a flocking state (strict motional consensus). In order to catch the main feature of motional consensus without sacrificing long simulation times, we take ${\phi _{m} = 0.979}$ to be the standard for reaching motional consensus. ${\phi _{m} = 0.979}$, which means the system almost reaches flocking state, is large enough to ensure the validity of analysis of motional consensus below will not change. In simulation, we set ${L = 10}$. The total time steps for simulation are 1000, which is long enough for the system to reach motional consensus.

Considering the rules of velocity alignment in the standard Vicsek model, the update of the direction of velocity will be directly influenced by their neighbors. Particles with common neighbors will build correlation of their velocity, while there will be no correlation of the velocity among the particles without common neighbor. To quantify simply, here, we only consider the correlation of each pair of particles consisting of two particles.

**Figure 1:**
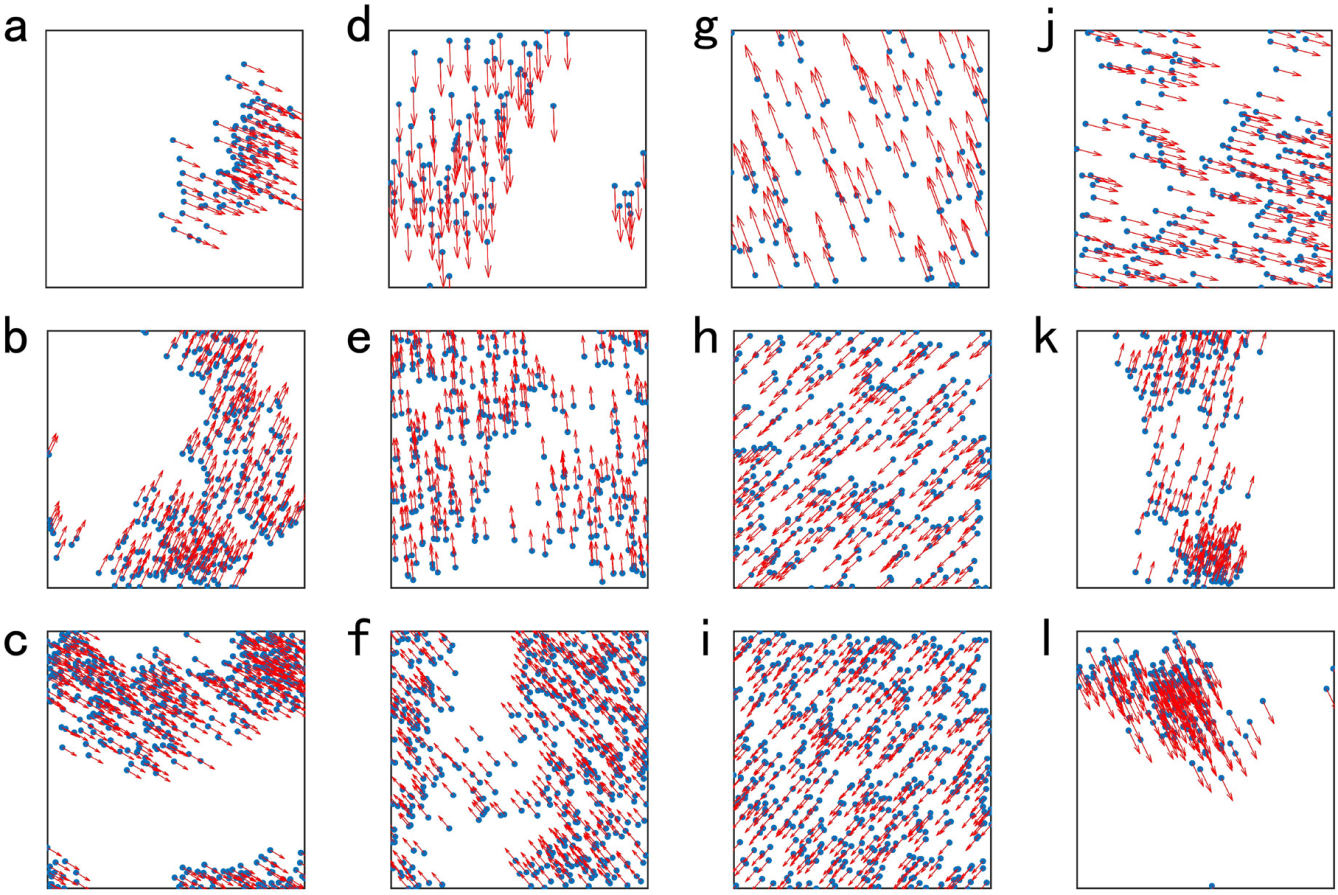
The movement pattern of the system reaching motional consensus for different interaction radius *r*, different number of particles *N* and different velocity *v*. The blue points denote the particles and the red arrows denote the direction of the velocity of the particles. ${v = 0.04}$ for (**a**)-(i). ${r = 0.8}$ for (**a**) ${N = 100}$, (**b**) ${N = 300}$ and (**c**) ${N = 500}$. ${r = 1.2}$ for (**d**) ${N = 100}$, (**e**) ${N = 300}$ and (**f**) ${N = 500}$. ${r = 5.0}$ for (**g**) ${N = 100}$, (**h**) ${N = 300}$ and (**i**) ${N = 500}$. ${r = 1.2}$ for (**j**) ${v = 0.01}$, (**k**) ${v = 0.16}$ and (**l**) ${v = 0.20}$. The value of other parameters for simulation are ${v = 0.04, L = 10}$ in (**a**)-(i) and ${N = 200, L =10}$ in (**j**)–(**l**).

When the system reaches motional consensus, they are found to have different movement patterns which show different spatial distribution and different degree of aggregation of all of the particles. As shown in Fig. 1, with the increasing of the interaction radius *r*, the spatial distribution of the particles is more uniform and the particles aggregate less closer. As the total number of particles increases, the particles are distributed more evenly and clustered closer together. The increase in velocity makes the spatial distribution of particles more uneven and the particles aggregate more closer.

To quantitatively study the movement pattern of the systems reaching motional consensus, we proposed a method with grid-based technique. Grid-based technique is widely used in many numerical approaches including Fast Multipole Method(FMM)^45^ and Multi-Particles Collision(MPC) etc^46^. FMM divides the space into different number of cells depending on the level of division. By analyzing the spatial relationship between the target cells and other cells, the interaction among particles in the target cell and the interaction of that with other cells can be obtained. Then the total interaction among the particles of the system can be evaluated. MPC introduces randomly shifted cells in the simulation of each time step. Evaluating the streaming and collision of the particles in each cell to obtain the position and velocity of the mass of the center of each cell. Then analyzing the dynamics of the system by considering the interaction among all of the cells.

**Figure 2:**
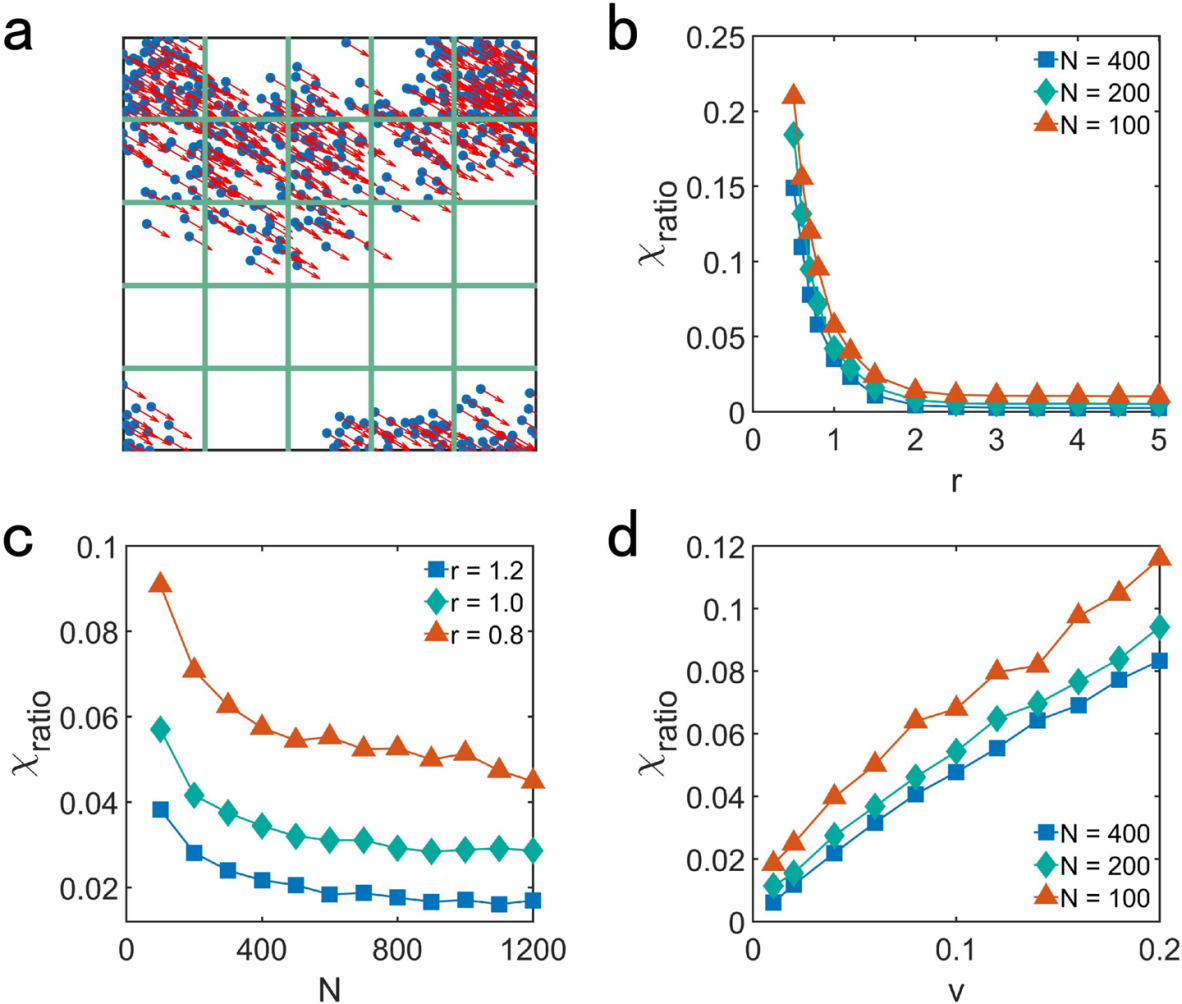
(**a**) Scheme of dividing the two-dimensional ${L \times L}$ space into 25 square grids with equal size. The normalized variance of the ratio of the number of particles in each grid to total number of the particles ${\chi _{ratio}}$ , showing the spatial distribution of the particles, as a function of (**b**) interaction radius *r* for ${v = 0.04, L = 10}$, (**c**) total number of particles *N* for ${v = 0.04, L = 10}$ and (**d**) the velocity of the particle *v* for ${r = 1.2, L = 10}$. The value of each data point in (**b**), (**c**) and (**d**) is the average of 200 different realizations.

Both of the method mentioned above can improve the efficient of the simulation. But they are more suitable to solve the interaction among the particles. Here, we aim to catch the feature of the movement pattern of the system and our method with grid-based technique is simple and efficient. As shown in Fig. 2a, we divide the two-dimensional ${L \times L}$ space into *G* grids, where ${G = 25}$ here. Then we investigate the normalized variance of the ratio of the number of particles in each grid ${N_{i}}$ to total number of the particles *N*.

The rationality of current grid selection is discussed in the Supplementary Information. The ratio of the number of particles in each grid ${N_{i}}$ to total number of the particles *N* is obtained as follows

7 $$\begin{aligned} R_{i} = \frac{N_{i}}{N} \end{aligned}$$

The normalized variance of the ratio of ${N_{i}}$ to *N* is

8 $$\begin{aligned} \chi _{ratio} = \frac{\sigma }{\sigma _{max}} \end{aligned}$$

and ${\sigma }$ is the variance of the ratio of ${N_{i}}$ to *N*

9 $$\begin{aligned} \sigma = \frac{ \left( \sum _{i} (R_{i} - {\bar{R}})^{2} \right) }{G} \end{aligned}$$

where ${{\bar{R}}} =\left(\sum _{i} R_{i}\right)/G = 1/G$ is the average of ${R_{i}}$. ${\sigma _{max}}$ denotes the maximum of the variance of the ratio of ${N_{i}}$ to *N* when all of the particles aggreagate in the same grid, which is

10 $$\begin{aligned} \sigma _{max} = \frac{1}{G}\left( 1-\frac{1}{G} \right) ^{2} + \left( \frac{1}{G} \right) ^{2G-1} \end{aligned}$$

The normalized variance ${\chi _{ratio}}$ will be 1 when all of the particles aggregate in the same grid, while ${\chi _{ratio} = 0}$ when all of the particles are uniformly distributed in *G* grid.

As Fig. 2b–d shown, the increase of *r* or *N* and the decrease of *v* leads to smaller value ${\chi _{ratio}}$ which means the more uniform spatial distribution of the particles (Supplementary Information 1).

**Figure 3:**
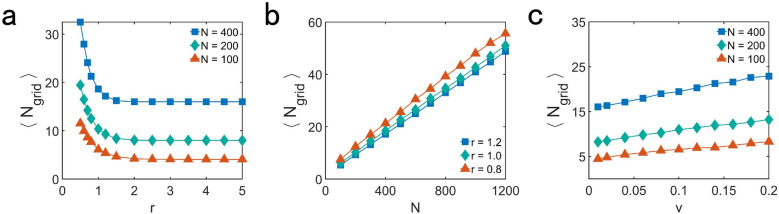
The average number of particles for the grid that occupied by particles ${\langle N_{grid} \rangle }$, showing the degree of aggregation of the particles, as a function of (**a**) the interaction radius *r* for ${v = 0.04, L = 10}$, (**b**) total number of particles *N* for ${v = 0.04, L = 10}$ and (**c**) the velocity of the particles *v* for ${r = 1.2, L = 10}$. The value of each data point is the average of 200 different realizations.

To quantify the degree of aggregation of the particles, we investigate the average number of particles for the grid that occupied by particles ${\langle N_{grid} \rangle }$. As Fig. 3a shown, with the increasing of *r*, the value of ${\langle N_{grid} \rangle }$ becomes smaller, which means the aggregation of the particles is less close. As shown in Fig. 3b,c, ${\langle N_{grid} \rangle }$ increases as *N* or *v* increase. The increase of *N* or *v* makes the particles aggregate more closer.

In order to understand the variation of the degree of aggregation of particles and the spatial distribution of the particles with different *r* and *N*, we study the common neighbors of particles. The common neighbors of particles are significant for the movement pattern of the particles by affecting the update of the velocity and position of particles. For noise-free cases, the movement pattern of particles when reaching motional consensus depends only on the initial state of all of the particles. Given the initial state, the state of the system is fixed after updating in each time step, because the update in each time step is not affected by noise.

**Figure 4:**
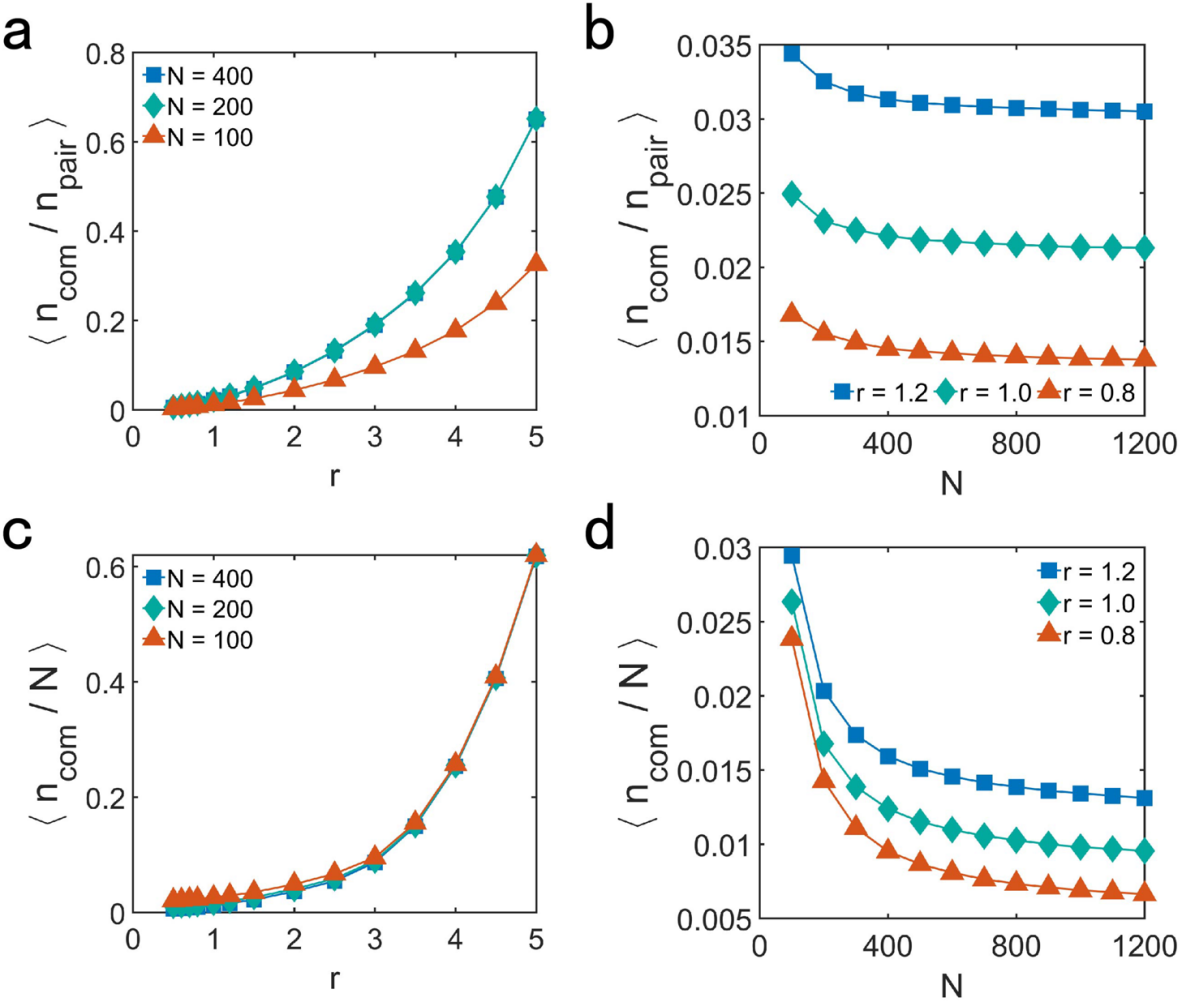
The average ratio of the number of common neighbors to the number of particles within the field of vision of pairs of particles ${\langle n_{com}/n_{pair} \rangle }$, showing the average strength of the correlation of velocity for each pair of particles, as a function of (**a**) *r* and (**b**) *N*. The average ratio of the number of common neighbors to the total number of the particles N in initial state ${\langle n_{com}/N \rangle }$, showing the average strength of the correlation of velocity between pairs of particles and all of the particles, as a function of (**c**) *r* and (**d**) *N*. In (**a**)–(**d**), the value of other parameters for simulation are ${v = 0.04, L =10}$. The value of each data point is the average of 200 different realizations.

For the initial state, we first pay attention to the average ratio of the number of common neighbor ${n_{com}}$ to the number of particles within the field of vision of pairs of particles ${n_{pair}} = n_{A} + n_{B} - n_{com}$, where ${n_{A}}$ and ${n_{B}}$ are the number of neighbors of particle A and B, which are any two of the *N* particles. ${\langle n_{com} / n_{pair} \rangle }$ reveals the average strength of the correlation of velocity for each pair of particles. As Fig. 4a,b shown, the decrease of *r* or the increase of *N* leads to weaker correlation of velocity for each pair of particles.

Because the motional consensus of the system is not only affected by the pairwise correlations of the velocity, but also related to all of the particles, we also investigate the average ratio of the number of common neighbor ${n_{com}}$ to the total number of the particles *N* in initial state. As shown in Fig. 4c,d, with the decreasing of *r* or the increasing of *N* there is weaker correlation of velocity between pairwise particles and all of the particles.

What have been analyzed about Fig. 4 shows that increasing *N* and decreasing *r* will weaken correlation of velocity among particles. Weaker correlation of velocity among particles makes it more difficult for particles to reach motional consensus. The particles will keep moving in their direction respectively until the correlation between their velocity is large enough to enable them to move in the almost same direction. In order to build stronger correlation of their velocity, particles will move more closer, resulting in larger degree of aggregation when the system reaches motional consensus.

There is a different mechanism for the effect of velocity to the degree of aggregation of particles. For small velocity, particles move slowly to close in order to reach motional consensus by building stronger correlation of their velocity. Because particles move slowly, they are more sensitive to the boundary that whether they can reach motional consensus or not. With the increasing velocity, particles move fast and are insensitive to the boundary that whether they can reach motional consensus or not, which leads to larger aggregation of the particles.

### Cases with noise

**Figure 5:**
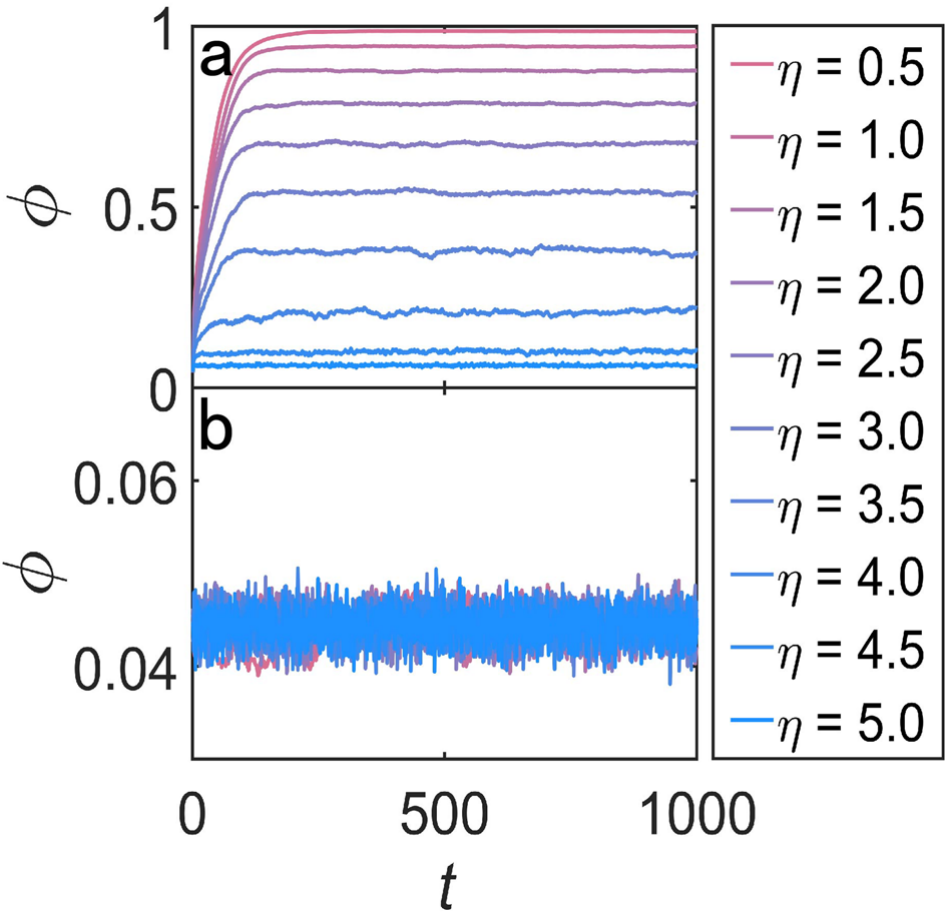
Order parameter ${\phi }$ as a function of time steps for different values of ${\eta }$ (**a**) With the effect of both velocity alignment and noise. (**b**) Just with the effect of noise. In both (**a**) and (**b**), the value of other parameters for simulation are ${v = 0.04, L = 10, N = 400, r = 1.0}$.

The rules of velocity alignment make the velocity of all of the particles unified, while the noise disturbs the motional consensus. For the motion of the particles, the restriction of velocity alignment will be weakened by the effect of noise, which makes the motional consensus more difficult or even impossible to reach. The temporal evolution of the order parameter for various strengths of noise are shown in Fig. 5a.

There is perturbation of order parameter when the state of the system can be thought to be steady. In order to quantify the value of order parameter when the system is nearly steady, we take the average of order parameter from 500 steps to 1000 steps as the order parameter of the system in the nearly steady state. With the increasing of ${\eta }$, the steady value of the order parameter is smaller, which means the less unified of the motion of all of the particles. Figure 5b shows the order parameter for different values of ${\eta }$ in the absence of velocity alignment. It is impossible to reach motional consensus even in the case of small ${\eta }$.

In order to quantify the effect of noise on motional consensus, we investigate the difference of order parameter ${\Delta \phi }$ between two cases as follows

11 $$\begin{aligned} \Delta \phi = \phi _{va} - \phi _{vn} \end{aligned}$$

where ${\phi _{va}}$ denotes the steady value of order parameter in the cases that the motion of particles is only restricted by velocity alignment and ${\phi _{vn}}$ denotes the steady value of order parameter in the cases that the motion is affected by both velocity alignment and noise.

**Figure 6:**
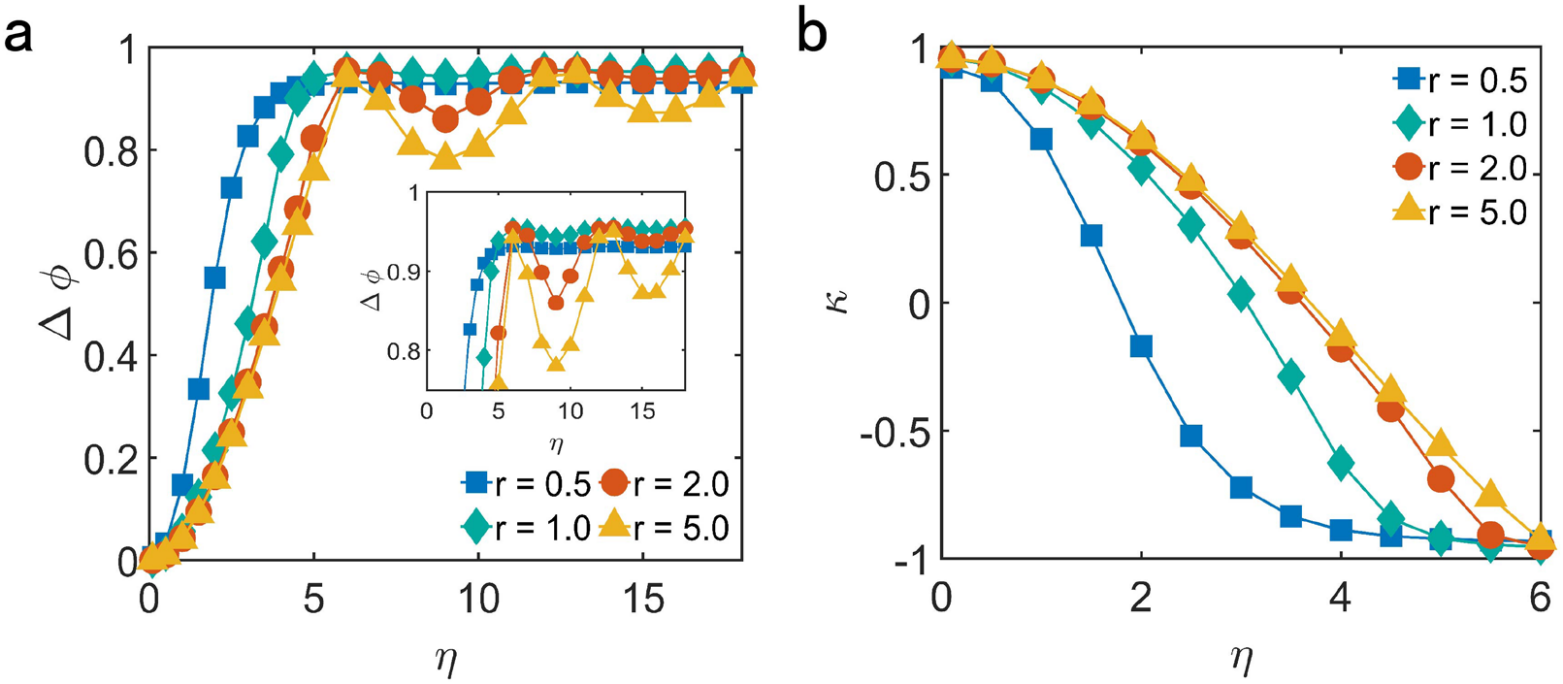
(**a**) The difference of order parameter between the cases that the update of the motion of particles is affected by both velocity alignment and noise and the cases that the motion is just affected by noise ${\Delta \phi }$, showing the effect of noise on motional consensus, as a function of ${\eta }$ for different interaction radius *r*. Inset: ${\Delta \phi }$ in the range of [0.7, 1] for clearly showing the variation of it as the ${\eta }$ increases. (**b**) The competition between velocity alignment and noise ${\kappa }$ as a function of ${\eta }$ for different interaction radius *r*. The value of each data point is the average of 200 different realizations. In both (**a**) and (**b**), the value of other parameters for simulation are ${v = 0.04, N = 400, L =10}$.

As shown in Fig. 6a, the difference of order parameter ${\Delta \phi }$ increases with the increasing of the value of ${\eta }$ for different interaction radius *r*, which shows the larger effect of noise on motional consensus.

To compare the influence of velocity alignment and noise on reaching motional consensus and quantify the competition between the effects of velocity alignment and noise, we defined the difference of the difference of the order parameter ${\kappa }$ which is shown as follows

12 $$\begin{aligned} \kappa = \Delta \varphi - \Delta \phi \end{aligned}$$

where ${\Delta \varphi = \phi _{va} - \phi _{n}}$ denotes the difference of order parameter between the cases that the update of the motion of particles is affected by both velocity alignment and noise and the cases that the motion is just affected by noise. As shown in Fig. 6b, the value of ${\kappa }$ will change from positive to negative as ${\eta }$ increases, which means the increasing of ${\eta }$ improves the effect of the noise to the motion of the particles and the effect of velocity alignment becomes more and more weak comparing that with the noise. When ${\kappa = 0}$, velocity alignment and noise affect motional consensus equally.

**Figure 7:**
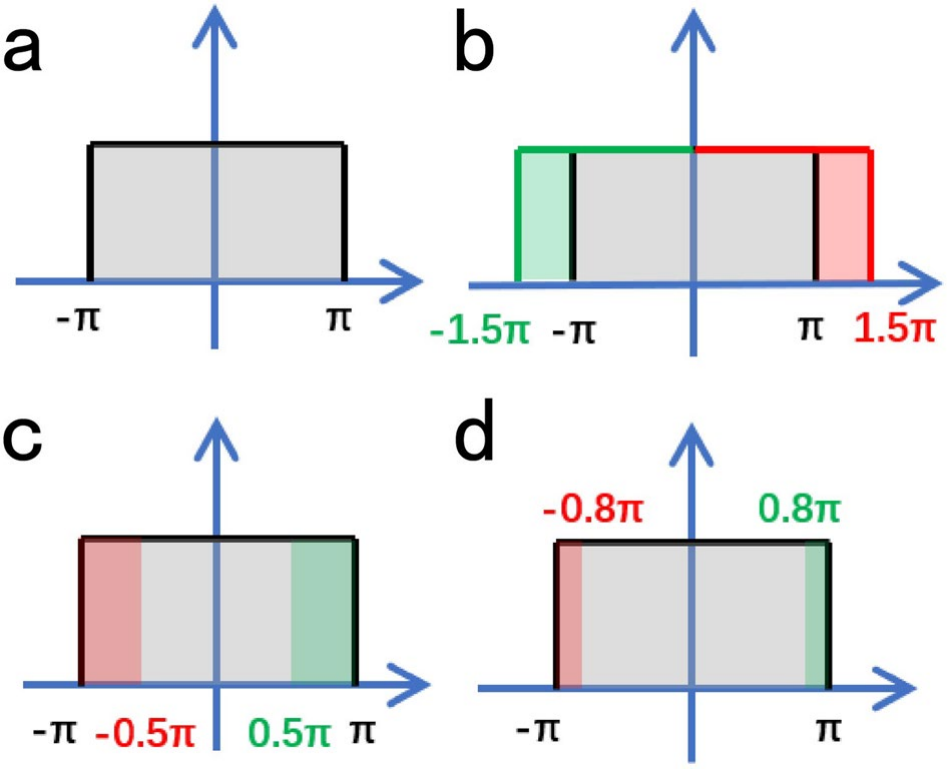
Scheme of the probability distribution of the direction of all of the particles. (**a**) The grey area denotes ${\Delta \theta }$ is in the range of ${[-\pi , \pi ]}$. (**b**) The probability distribution of ${\Delta \theta }$ when ${\Delta \theta \in [-1.5\pi , 1.5\pi ]}$. The green area denotes the probability in the case of ${\Delta \theta \in [-1.5\pi , \pi ]}$ and the red area denotes the probability when ${\Delta \theta \in [\pi , 1.5\pi ]}$. (**c**) The probability distribution of ${\Delta \theta }$ when unifing all of the value from ${[-1.5\pi , 1.5\pi ]}$ to ${[-\pi , \pi ]}$. (**d**) The probability distribution of ${\Delta \theta }$ when unifing all of the value from ${[-1.2\pi , 1.2\pi ]}$ to ${[-\pi , \pi ]}$. The red area and the green area denotes the probability when ${\Delta \theta \in [-1.2\pi ,\pi ]}$ and ${\Delta \theta \in [\pi , 1.2\pi ]}$ respectively before unifying and they denote ${\Delta \theta \in [-\pi , -0.8\pi ]}$ and ${\Delta \theta \in [0.8\pi , \pi ]}$ after unifying.

We also observed that the variation of ${\Delta \phi }$ is not monotonous when the value of ${\eta }$ is larger than 6. When ${\eta = 6}$, ${\Delta \theta \in [-3.0, 3.0]}$, which is close to ${[-\pi , \pi ]}$, the probability of all the direction of velocity effected by noise is almost equal as shown in Fig. 7a.

Because of the periodicity of the angle denoting the direction of velocity, the probability distribution of the noise will be changed when ${\eta > 6}$. For example, as shown in Fig. 7b,c, when ${\eta = 3\pi }$ which means ${\Delta \theta \in [-1.5\pi , 1.5\pi ]}$, the probability of ${\Delta \theta \in [-1.5\pi , -\pi ]}$ and ${\Delta \theta \in [\pi , 1.5\pi ]}$ will be unified in the probability of ${\Delta \theta \in [-0.5\pi , -\pi ]}$ and ${\Delta \theta \in [0.5\pi , \pi ]}$ respectively, which changing the probability distribution of ${\Delta \theta }$. As shown in Fig. 7c,d, in the cases of ${\eta \in [2\pi , 3\pi ]}$, the more the ${\eta }$ larger than ${2\pi }$, the less the ${\Delta \phi }$ will be. When ${\eta \in [3\pi , 4\pi ]}$, the probability unified in ${[-\pi , \pi ]}$ makes the probability distribution of ${\Delta \theta }$ more uniform, which leads to the increment of ${\Delta \phi }$.

**Figure 8:**
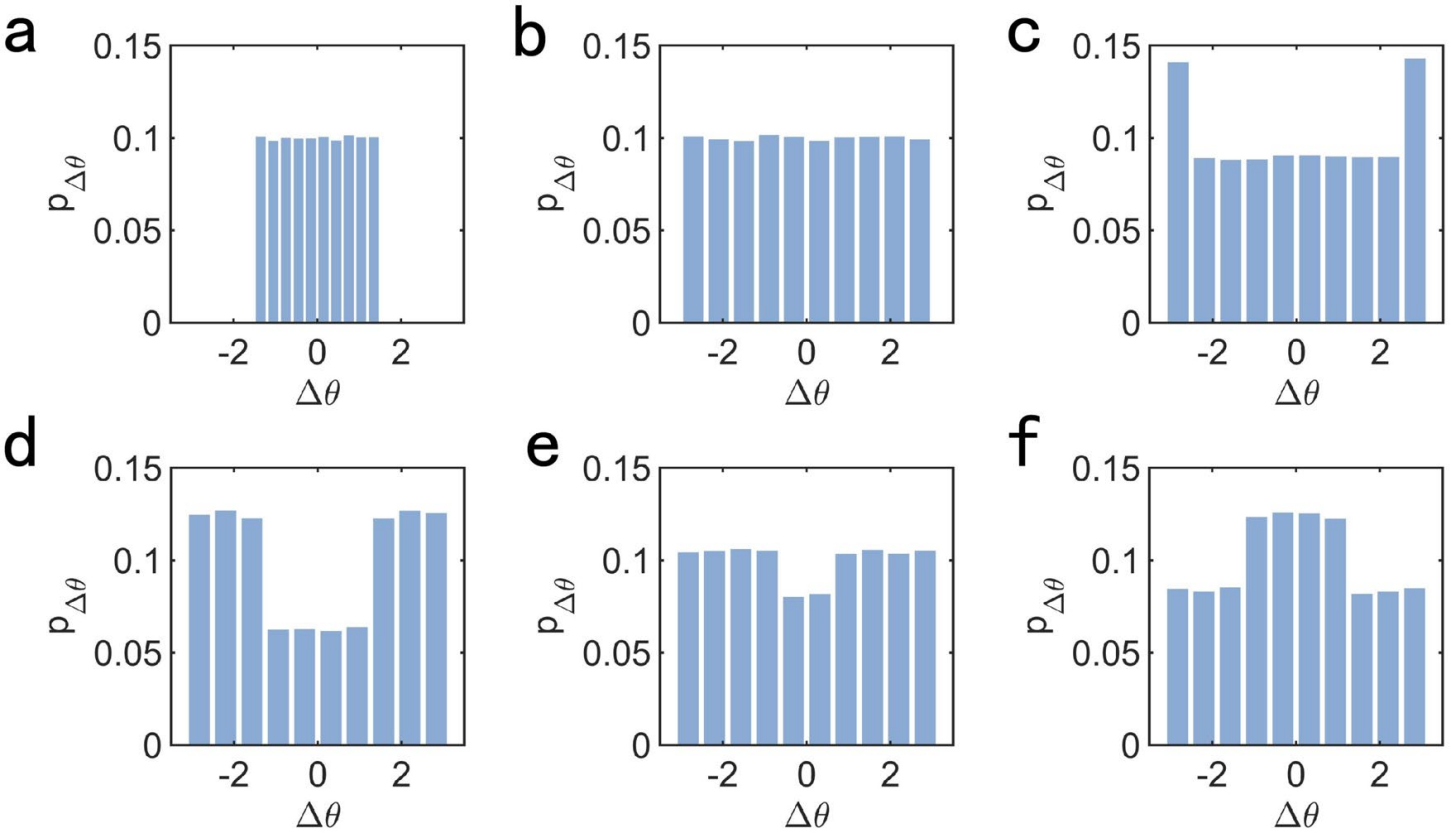
Probability distribution of noise ${P_{\Delta \theta }}$ when (**a**) ${\eta = 3.0}$, (**b**) ${\eta = 6.0}$, (**c**) ${\eta = 7.0}$, (**d**) ${\eta = 10.0}$, (**e**) ${\eta = 12.0}$, (**f**) ${\eta = 15.0}$.

In order to confirm our analysis of the reason of the non-monotonic variation of ${\Delta \phi }$ when ${\eta > 6}$, we investigate the probability distribution of noise with different value of ${\eta }$. As shown in Fig. 8, different values of ${\eta }$ lead to different probability distribution of ${\Delta \theta }$, which affect the value of ${\Delta \phi }$.

This is consistent with the above analysis of noise. As shown in the inset of Fig. 7a, the degree of the non-monotonic variation about the difference of order parameter is larger with the increasing of the interaction radius *r*. This is because the larger interaction radius improves the effect of velocity alignment on motional consensus, which makes the competition between velocity alignment and noise more intense. This makes the difference of order parameter ${\Delta \phi }$ more sensitive to the change of the distribution of noise.

## Conclusion

In conclusion, we have studied the motional consensus of self-propelled particles in both the noise-free cases and the cases with noise by standard Vicsek model.

For the noise-free cases, we have proposed a method to quantitatively describe the spatial distribution of the particles by divided the two-dimensional space into some grid with equal size and count the normalized variance of the ratio of ${N_{i}}$ to *N*. It is found that the smaller *r* or larger *N* builds weaker correlation of the velocity among particles, which leads to larger degree of aggregation of the particles when the system reaches motional consensus.

For the cases with noise, we have quantitatively analyzed the competition between the effects of velocity alignment and noise on the degree of motional consensus. The results show that the non-monotonic variation of the effect of noise on motional consensus result from the non-uniform probability distribution of the noise.

Collective behaviors of active systems present various patterns. Pattern formation of active systems may be studied by generalizing the Smoluchowski aggregation theory which focus on the growth and distribution of clusters for passive systems^47^. Bridging the Vicsek model(particles-based model) and the theroy proposed by Tu and Toner based on hydrodynamics^48^ is also an interesting perspective of study about collective behavior of active systems. As for the further studies concerning the collective behavior of active systems, our study may be useful for exploring the basic principle of collective motion.

## Supplementary Information

Supplementary Information.

## Data Availability

The data presented in this study are available on request from the corresponding author.
