# Supplementary material for: Motional consensus of self-propelled particles
Source: Sci Rep. 2023 May 20;13:8169. doi: 10.1038/s41598-023-35238-w (PMC10199942; doi:10.1038/s41598-023-35238-w)
Supplement: Supplementary file 1 — Supplementary Information. [file 41598_2023_35238_MOESM1_ESM.pdf]

1. The number of grids on dividing the $L \times L$ space

In order to quantify the degree of aggregation of the particles, reaching motional consensus, in the two dimensional space, we divide the $L \times L$ space with G grid. We investegate the effect of the number of grids G on quantifying the degree of aggregation and quantify the effect by the difference between the maximum value of χ and its minimum value, which is

$$\Delta\chi = \chi_{max} - \chi_{min} \tag{1}$$

As shown in Fig. S1, the variation of the difference of χ is non-monotonic. When the number of grids is small, the divison of space is so rough that the number of particles in each grids is in a greater contingency. While the difference between χ_{max} and χ_{min} is too small to show the variation of χ_{area} with the variation of the interaction radius r. In considering what have been mentioned above, we chooses the number of grids with medium value of $\Delta\chi$, which is $G = 25$.

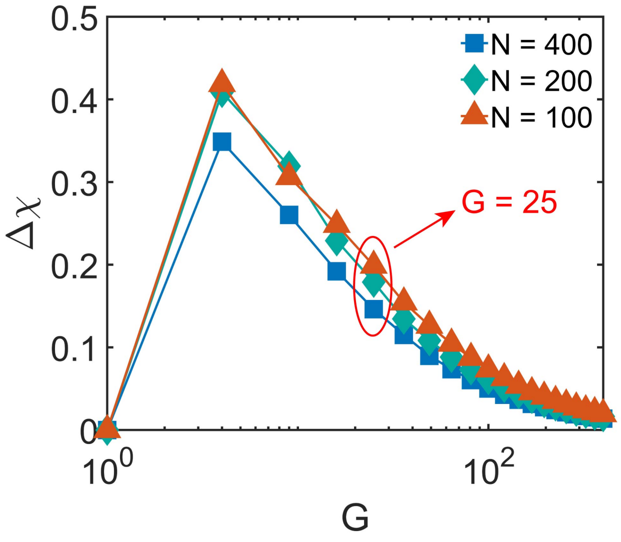

Figure S1: The difference of the variance of the ratio of the number of particles in each grids to the total number of particles as a function of the number of grids
